# Recent Advances in Adsorptive Nanocomposite Membranes for Heavy Metals Ion Removal from Contaminated Water: A Comprehensive Review

**DOI:** 10.3390/ma15155392

**Published:** 2022-08-05

**Authors:** Fouad Damiri, Swetha Andra, Nagavendra Kommineni, Satheesh Kumar Balu, Raviteja Bulusu, Amira A. Boseila, Damilola O. Akamo, Zubair Ahmad, Farhat S. Khan, Md. Habibur Rahman, Mohammed Berrada, Simona Cavalu

**Affiliations:** 1 Laboratory of Biomolecules and Organic Synthesis (BIOSYNTHO), Department of Chemistry, Faculty of Sciences Ben M’Sick, University Hassan II of Casablanca, Casablanca 20000, Morocco; 2 Department of Chemistry, Rajalakshmi Institute of Technology, Chennai 600124, Tamil Nadu, India; 3 Center for Biomedical Research, Population Council, New York, NY 10065, USA; 4 Department of Oral Pathology, Saveetha Dental College and Hospitals, Saveetha Institute of Medical and Technical Sciences, Chennai 600077, Tamil Nadu, India; 5 Department of Pharmaceutical Sciences, Florida A&M University, Tallahassee, FL 32307, USA; 6 Department of Pharmaceutics, National Organization for Drug Control and Research (NODCAR), Cairo 12611, Egypt; 7 Department of Pharmaceutics and Industrial Pharmacy, Faculty of Pharmacy, Sinai University, Sinai 41636, Egypt; 8 The Bredesen Center for Interdisciplinary Research and Graduate Education, University of Tennessee, Knoxville, TN 37996, USA; 9 Unit of Bee Research and Honey Production, Faculty of Science, King Khalid University, P.O. Box 9004, Abha 61413, Saudi Arabia; 10 Biology Department, College of Arts and Sciences, Dehran Al-Junub, King Khalid University, P.O. Box 9004, Abha 61413, Saudi Arabia; 11 Department of Global Medical Science, Wonju College of Medicine, Yonsei University, Wonju 26426, Korea; 12 Faculty of Medicine and Pharmacy, University of Oradea, P-ta 1 Decembrie 10, 410087 Oradea, Romania

**Keywords:** nanocomposite membranes, nanomaterials, heavy metals removal, water treatment, adsorption

## Abstract

Water contamination is one of the most urgent concerns confronting the world today. Heavy metal poisoning of aquatic systems has piqued the interest of various researchers due to the high toxicity and carcinogenic consequences it has on living organisms. Due to their exceptional attributes such as strong reactivity, huge surface area, and outstanding mechanical properties, nanomaterials are being produced and employed in water treatment. In this review, recent advances in the use of nanomaterials in nanoadsorptive membrane systems for wastewater treatment and heavy metal removal are extensively discussed. These materials include carbon-based nanostructures, metal nanoparticles, metal oxide nanoparticles, nanocomposites, and layered double hydroxide-based compounds. Furthermore, the relevant properties of the nanostructures and the implications on their performance for water treatment and contamination removal are highlighted. The hydrophilicity, pore size, skin thickness, porosity, and surface roughness of these nanostructures can help the water permeability of the nanoadsorptive membrane. Other properties such as surface charge modification and mechanical strength can improve the metal adsorption effectiveness of nanoadsorptive membranes during wastewater treatment. Various nanocomposite membrane fabrication techniques are also reviewed. This study is important because it gives important information on the roles of nanomaterials and nanostructures in heavy metal removal and wastewater treatment.

## 1. Introduction

Heavy metal discharge into wastewater from industry is a major environmental concern. The accumulation of these heavy metal contaminants in wastewater is particularly harmful to both humans and aquatic life; hence, the food chain process is negatively impacted [1,2]. Life depends on water, as it is a fundamental resource on earth, and people and other organisms need it to survive. Despite the fact that water is the most basic requirement, activities undertaken in the name of economic development have contaminated and made it hazardous for life in both water and on land [3]. Heavy metals have been indicated to be cancerous, and they have been shown to damage the kidneys as well as the lungs, mental health, central nervous system, and various other organs. Heavy metal removal from water is therefore critical, and it has garnered much attention in recent years. Several solutions to this issue have been offered, including electrochemical treatment, co-precipitation, membrane filtration, and adsorption. Furthermore, it is frequently the case that different removal methods are coupled in order to obtain better results. Adsorption is among the most extensively used of the aforementioned strategies, owing to the low cost and simplicity of operation [4,5,6]. Numerous adsorbents, including zeolite, clay minerals, carbon foam, activated carbon, polymers, biochar, and fly ash, are being used to remove heavy metals through adsorption. Regarding the economic feasibility and environmental significance, carbohydrate biopolymers such as cellulose, chitosan, and alginate have received considerable attention over the last twenty years as alternative efficient adsorbents [7,8]. This is due to their outstanding advantages including their natural abundance, eco-friendliness, biodegradability, ease of modification, and low-cost of production. Several studies have been conducted regarding adsorbents with high adsorption capacity that may be prepared by integrating a natural polymer in the matrix in order to prepare adsorbent from such materials. Various types of natural, modified natural, and synthetic polymers have been used [9,10,11,12,13].

However, scientific progress has expanded notice of water contaminant types and reawakened the necessity for comprehensive water treatment. Oxyanions (or oxoanions) (As, V, B, W, and Mo) are formed by a number of redox-sensitive metalloids and metals, including titanium dioxide, iron oxides, manganese dioxide, aluminum oxides, and numerous oxide minerals [14,15]. Due to their toxicity, non-degradability, and movement in aquatic habitats, these species are detrimental to living organisms. Adsorption is among the most straightforward, cost-effective, and inexpensive water treatment methods. Since metal-oxide-based adsorbents have a range of functional groups on their surface, they have been utilized extensively in ion sorption [14]. Among other adsorbents, carbon and carbon derivatives are widely used adsorbents, owing to their higher efficiency in adsorption. Physical characteristics such as large surface area and ease of chemical modification contribute to their remarkable performance, making them globally accepted material for the removal of a broad range of pollutants [6,16]. Activated carbons, on the other hand, have a variety of problems that limit their use. They are expensive to make, difficult to decompose, and the process of regenerating them is time-consuming and inefficient. As a result, there has been a lot of research into low-cost adsorbents [17]. In addition to low-cost adsorbents, adsorptive membranes are also considered as an efficient absorbent material because of their functional adsorption groups as well as structural characteristics that support the membrane’s ability to remove heavy metals [18]. Some other techniques applied to remove heavy metals from water are through adsorptive columns and membrane systems. Though these fall under the cost-effective category, they possess great disadvantages over novel adsorptive membranes and nanoadsorptive membranes. Even though adsorptive columns have the capability to remove metals from water, repeated use of columns is required for effective purification. The majority of membranes in membrane systems is composed of polymers (adsorptive removal of heavy metals from water using sodium titanate nanofibers loaded onto GAC fixed-bed columns). Another difficulty is that the polymer matrix encloses part of the active sites for adsorption, reducing their surface area and impairing the kinetics of adsorption. Hydrophilic polymers can be used to remedy this problem; however, they are incompatible with melt blending, which is more ecologically friendly. However, given the arduous stirring necessary for adsorption, the durability of membrane-based adsorbents constructed from hydrophilic polymers is still in doubt. Polymeric membranes have been the most common membranes used in water treatment. Membranes have the potential to remove compounds from aqueous streams, lowering the number of pollutants in wastewater. The presence of numerous contaminants and a large number of molecules, as well as ions, in polluted waters complicate water purification and separation by membranes [19].

In recent years, nanotechnology paired with known procedures has proved its usefulness in the water sector in eliminating pollutants and lowering toxicity and is emerging as a viable alternative to conventional wastewater treatment [20]. Nanomaterials outperform typical materials in terms of physicochemical properties such as intra-particle diffusion length, high surface area, mild temperature fluctuations, good surface chemistry adsorption sites, and pore size distribution. Several studies have been conducted to investigate the removal of heavy metals from wastewater using various nanoadsorbents [21]. A range of nanoadsorbents, including carbon-based compounds, zeolites, metal organic frameworks (MOFs), metals, and metal oxides has also been employed to separate heavy metals from bodies of water [22]. Despite having a larger adsorption ability for heavy metal ions, nanoscale materials are more susceptible to agglomeration owing to Van der Waals interactions, lowering their adsorption capacity [23]. Considering that nanomaterials are difficult to separate from aqueous medium, they are also inappropriate for use in fixed column beds or other flow-through systems, owing to their low mechanical strength and considerable pressure drop [24]. To overcome these limitations, imbedding nanoadsorbents into highly permeable material such as polymeric membranes is a potential approach. Adsorptive membranes were created by embedding a potential nanoadsorbent inside polymeric membranes, combining the benefits of adsorption and membrane separation. When heavy metal-contaminated water diffuses across membranes, these nanoadsorbents may absorb the heavy metal ions efficiently, resulting in the production of clean permeate [25]. The current study focused on recent developments in adsorptive membranes for heavy metal ion removal. The fabrication method and process, as well as the obstacles and prospects are all discussed.

## 2. Nanomaterials in Wastewater Treatment

### 2.1. Carbon-Based Nano Adsorbents

Carbon-based nanomaterials, including carbon fibers, carbon nanotubes (CNT), activated carbons, graphene, graphene oxide, and other graphene-related materials are widely regarded as among the most effective adsorbents for extracting organic and inorganic contaminants from wastewater. Typically, these materials have a large specific surface area, strong chemical stability in acid/alkaline conditions, enhanced mechanical and thermal stability, and high porosity [26,27]. Although carbon nanomaterials have many advantages, the routinely employed ones, such as graphene and CNT, have minimal functionalization and inadequate dispersibility in aqueous conditions, leading to low adsorption [28]. In order to increase the solvent dispersion and adsorption properties of these nanomaterials, innovative carbon nanocomposite materials have been produced by modifying the surface of CNTs or graphene and inserting additional active components on their surfaces [29,30].

Many research studies have shown that graphene oxide (GO) and reduced graphene oxide (rGO) have promising adsorption capabilities in the cleanup of heavy metal ions, organic pollutants, and pharmaceutical toxins [31,32,33]. Gupta et al. demonstrated that rGO possesses an excellent surface area and a significant number of voids, allowing it to effectively remove malachite green dye from wastewater [34,35].

### 2.2. Metal-Based Nano Adsorbents

Nanoparticles (NPs) derived from iron oxide are commonly employed as heterogeneous catalysts owing to their unique magnetic and physicochemical characteristics. Controlling the features of nanoparticles (such as particle shape, size, etc.) and incorporating more nanoparticles into the system might increase the catalytic efficiency of iron oxide nanoparticles. It has been shown that iron oxide nanoparticles are effective adsorbents for removing heavy metals from water sources. For instance, nanohematite showed a good absorbent ability to remediate spiced tap water ionized metal [36,37].

Another type of metal-based nanoadsorbent is titanium dioxide (TiO_2_) nanoparticles. TiO_2_ nanoparticles showed high capability for Cu(II), Pb(II), Zn(II), Ni(II), and Cd(II) adsorption [38].

Noble metals such as gold (Au), platinum (Pt), silver (Ag), and palladium (Pd) have high energies of ionization and as a consequence poor oxidation potential because of their short atomic size [39]. Noble metal nanoparticles were stabilized by surfactants and polymers [40]. Ag and Au nanoparticles were used in checking low levels of organic contaminants due to their distinctive visual properties. Additionally, bimetallic nanoparticles Au/Pt, Ag/Au, or Ag/Pt showed a high capacity for the sensing, monitoring, and photocatalysis of trace pollutants [41].

### 2.3. Hydrogels

Hydrogels are three-dimensional, cross-linked, and elastic polymer networks with hydrophilic groups such as hydroxyl, amide, and carboxyl that expand in water and biological fluids [42,43]. Numerous attempts have been undertaken in the field of wastewater treatment using hydrogels. In the majority of studies, research concentrated on the capacity of hydrogels to remove organic poisonous dyes and inorganic hazardous heavy metal ions, with a current emphasis on emerging contaminants [42,44].

Using UV-polymerization, Jinxiang Gao et al. created a bio-inspired cellulose paper–poly(amidoxime) composite hydrogel. Due to its improved hydrophilicity, strong hydraulic/ionic conductivity, and broad-spectrum antibacterial activity, this hydrogel had a very efficient uranium capture capacity of up to 6.21 mg.g^−1^ for WU/W dry gel and 12.9 mg.g^−1^ for WU/W poly(amidoxime) in saltwater for six weeks [45].

Liu et al. reported that massive uranium recovery from seawater is a vital project to meet the long-term need for worldwide nuclear power, but it is still very difficult to produce high-efficiency adsorbents with good mechanical properties. In this study, a novel nanoclay–poly(amidoxime) (NC–PAO) double-network (DN) composite hydrogel adsorbent with high-efficiency, high strength, and rapid uranium extraction capacity was investigated [46].

### 2.4. Nano-Sponges

Nano-sponges are characterized by the presence of cavities and mesh-like/colloidal structures containing solid nanomaterials. They are suitable for capturing various materials [47,48]. Moreover, nanosponges can be modified and or functionalized using different nanoparticles (TiO_2_, carbon nanotubes, Ag, etc.) for the elimination of organic/inorganic contaminants from wastewater [49,50]. Nano-sponge-based cyclodextrin showed good removing capacities of dissolved organic carbon from water (~84%) [20]. Additionally, zeolite-modified nanosponges showed promising capacities for removing nitrate in contaminated water [51,52].

### 2.5. Nanocomposites

Mohamed (2019) showed that a ternary α-Fe2O3/GO/WO3 nanocomposite enhances the photocatalytic degradation of crystal violet (CV) and methylene blue (MB) contaminants in water [36,53].

Additionally, the removal of fluoroquinolones (lomefloxacin, ciprofloxacin, and norfloxacin) from wastewater was examined using a nickel ferrite nanocomposite modified with L-cysteine-linked 3-glycidyloxypropyltrimethoxysilane. The carboxyl groups of the L-cysteine molecule acted as the removing target moiety for fluoroquinolones [54].

Magnetic oxide nanoparticles and hybrid carbon nanomaterials outperformed unmodified materials in terms of adsorption efficiency [55], which was ascribed to the active engagement of the metal oxide’s oxygen functional groups in the adsorption of cationic water contaminants [35,56,57].

### 2.6. Layered Double Hydroxide (LDH)-Based Materials

Layered double hydroxides (LDHs) are classed as anionic two-dimensional clays with distinctive layered structures and the chemical configuration of the inorganic layers exhibiting intriguing potential for dispersing many active sites [58,59,60]. Mubarak et al. produced Mg/Fe-LDH nanospheres comprising two metallic baseline materials, Mg^2+^ and Fe^3+^ [61]. They also prepared its oxidized phase in the form of Mg/Fe-based layered double oxide (LDO) nanospheres and utilized them to remove pollutants from wastewater, including oxy-anionic heavy metals (AsO_4_^3−^ and Cr_2_O_7_^2−^) and anionic organic dyes (such as methylene blue and Congo red). In another investigation, an Fe_3_O_4_/GO/LDH adsorbent was produced to remove Pd(II) and 2,4-dichlorophenoxyacetic acid from an aqueous system [62]. In another study, Pb(II) and organic pollutant adsorption capacity was improved by the addition of graphene oxide to Fe_3_O_4_/LDH adsorbents [35]. Summary of nanoparticles used as adsorbents for water treatment are shown in Table 1.

## 3. Nanomaterials Used for Heavy Metal Removal

Water is one of the most significant natural resources on earth, as it is essential for the survival of all living organisms and the development of humankind. In combination with industrialization and urbanization, water demand is expanding quickly, and scarcity has arisen as a serious financial constraint. Moreover, polluted water, namely, heavy metal pollution in water, has become a major environmental problem on a global scale. Electroplating, mining chemical plants, metallurgy, domestic wastewater, and agriculture can introduce heavy metals into water. Heavy metals such as zinc, lead, mercury, copper, and other elements may build up in the food chain and constitute a serious health danger to people. Heavy metals can, for example, harm kidneys, the central nervous system, the lungs, and other organs. Consequently, eliminating heavy metal ions from water is vital and has received much attention. Accordingly, nanomaterials have now been shown to be a viable option for eliminating heavy metals from wastewater [4]. Heavy metals are expected to require special attention due to their human carcinogenic effects and negative environmental consequences [75,76]. The United States Environmental Protection Agency (US EPA) has determined maximum contamination levels (MCL) for numerous heavy metals above which they are deemed harmful for human consumption, with values of 0.01, 0.006, and 0.05 mg/L for cadmium, lead, and chromium, respectively [77]. Their ions have been discovered to be hazardous to aquatic organisms even at low concentrations. Chemisorption and oxidation, reduction, solvent evaporation, electrochemical treatment, membrane filtration, coagulation, reverse osmosis, solvent evaporation, filtration, rehabilitation, electro-dialysis, and adsorption are some of the technologies used to treat wastewater and remove heavy metals. The majority of these technologies produce minimal industrial impacts, since they are time-consuming, costly, and result in secondary pollutants. Adsorption is one of the most efficient and extensively employed technologies for wastewater treatment due to the utilization of widely accessible by-products and inexpensive substitutes, including bio-wastes, fly ash, chitosan [78], and chitin. Additionally, adsorption involves the application of nanostructures for the extraction of these toxic substances from water bodies. These nanomaterials have unique qualities that include nanoscale particle size, high aspect ratio, strong solvent mobility, high reactivity, excellent mechanical properties, high porosity, wettability, and hydrophobicity [79].

Recently, nanotechnology has been incorporated with the majority of conventional methods to increase the rate of heavy metal removal from water bodies. In the last several decades, nanomaterials have attracted interest as a way to create cost-effective, ecologically acceptable, and extraordinarily effective methods for removing organic and inorganic toxins from the environment. Most nanomaterials have properties that make them ideal for water treatment or purification. Nanomaterials have a high sorption capacity and selectivity, allowing them to efficiently remove metal ions [24,80].

### 3.1. Carbon-Based Nanostructures

In recent years, carbon-based nanostructures including carbon nanotubes (CNTs) and graphene and its derivatives, such as graphene oxide (GO), have been utilized extensively for the separation of heavy metals. This is because of the non-toxic nature of these nanostructures as well as their increased adsorption capacity [81,82]. These materials are discussed below.

#### 3.1.1. Graphene

Due to their remarkable properties, graphene-based nanomaterials have become some of the most studied substances in recent years. Graphene is a hexagonally organized, single sheet of sp^2^-hybridized graphite. When graphene is oxidized and exfoliated, graphene oxide (GO), a single-layer graphene with a high concentration of oxygen, is produced. GO’s usage in heavy metal adsorption has gained a great deal of attention due to its exceptional features, including an unusually high surface area and enormous amounts of oxygen-containing groups with considerable affinity for heavy metals, such as –COOH, –OH, and C=O groups. In addition, GO is known for its high hydrophilicity, which implies that it is very soluble in water [83].

Following the collapse of a tailings dam in the Brazilian state of Minas Gerais, Lebron et al. used a modified version of the Hummers method to produce GO, and they tested the method’s capacity for removing heavy metals from a sample of natural surface water. The results revealed that Al, Cu, and Mn had the highest clearance rates of all metals, at 90.2%. Despite this, prolonged water consumption of aluminum would represent a moderate danger. The findings support GO’s ability to adsorb heavy metals, and its efficacy was confirmed even in intricate media [84]. The porous rGO aerogels were effectively developed utilizing a simple hydrothermal approach without the need of any solid or aqueous activation agent. Adsorption studies revealed that the rGO aerogels performed well during the elimination process of Pb^2+^ and could be recycled at least four times [85]. Heavy metal ion removal has been attributed to a number of mechanisms, the most recent of which have been size exclusion, adsorption, and electrostatic contact. The efficiency of the GO-incorporated membrane to eliminate heavy metal ions is dependent on ionic strength, pressure, temperature, feed flow rate concentration, ionic dispersibility in solvent, solution–GO membrane interaction, and the contribution of chelating agents [86].

**Figure 1:**
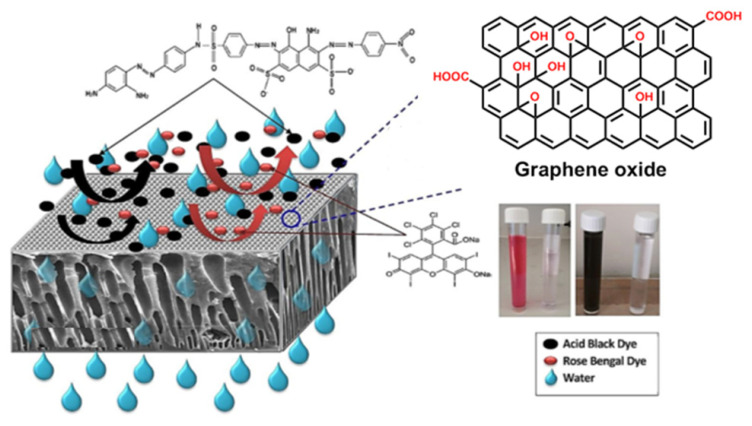
Dye removal using graphene oxide (GO) mixed matrix membranes [87].

Kadhim et al. modified the polyethersulfone (PES) membrane with graphene oxide nanoparticles (GO-NPs) to create mixed matrix membranes (MMMs) (Figure 1). This study employed two types of very poisonous dyes (rose Bengal and acid black) to examine the influence of GO on the performance of PES. Utilizing SEM, FTIR, AFM, water permeation flux, dye removal, and fouling, as well as studying the effect of GO-NPs on the structure, the performance and antifouling capabilities of the newly modified membrane were investigated. After adding 0.5% GO, the membrane’s contact angle was the smallest (39.21°), and its permeable flux was the greatest. For both dyes, the ultrafiltration (UF) membrane demonstrated a rejection rate greater than 99 percent. At a GO concentration of 0.5% by weight, the membranes exhibited the best antifouling properties. Long-term performance of the membrane produced from 0.5 wt.% GO with two dyes was significantly enhanced over 26 days compared to 14 days for the control membrane; hence, a greater flux may be maintained [87].

#### 3.1.2. CNTs

In addition to mesopores, the diameter of CNTs spans from 1 to several nm, and their specific surface area is between 150 and 1500 m^2^/g, making them an attractive choice for the adsorption-based removal of metal ions [88,89]. MWCNTs with chitosan and carrageenan membranes were produced and evaluated, and it was found that adding carrageenan and chitosan improved membrane characteristics and performance. The MWCNTs/chitosan exhibited significant permeability and mechanical and electrical properties. BET analysis showed a better surface area for MWCNTs/carrageenan. The MWCNTs/carrageenan–chitosan displayed improved thermal properties and excellent heavy metal removal efficiency, with up to 90% removal for several very hazardous metals at low pressure [90].

**Figure 2:**
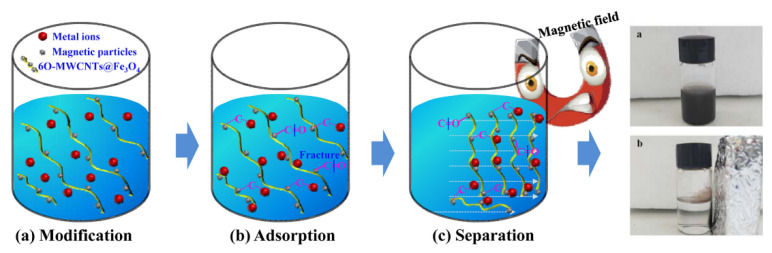
The mechanism of heavy metal ion absorption by 6O-MWCNTs@Fe_3_O_4_ in wastewater [91].

Wang et al. synthesized a novel form of recyclable adsorbent by oxidizing an enhancer and modifying it with magnetic nanoparticles (Figure 2). In addition to inheriting the benefits of multiwall carbon nanotubes (6O-MWCNTs), the novel adsorbent demonstrated unique magnetic properties and enhanced adsorption capacity, which is favorable for the magnetic separation and recovery of heavy metals. The adsorption findings revealed that multiwall magnetic carbon nanotubes (6O-MWCNTs@Fe3O4) had a strong performance for Pb(II) selective adsorption, with a maximum adsorption capacity of 215.05 mg/g, which is much more than the current adsorption capacity of the same kind of adsorbents [91].

Adam et al. (2021) created a simple and environmentally compatible material for eliminating heavy metal ions, including Hg^2+^, Pb^2+^, Cd^2+^, and Sn^2+^, by employing a unique metal–carbon-based composite such ZnFe_2_O_4_-carbon nanotubes (CNT). The carbon nanotubes were ground with ZnFe_2_O_4_ to produce a black homogenized solid ZnFe_2_O_4_-CNT sorbent. The results indicated that the following parameters are optimal for efficiently recovering the investigated metal ions from aqueous solution at a dosage of 20 mg/100 mL with pH 5 for the Hg^2+^ and Pb^2+^ ions, pH 6 and an adsorbent dose of 50 mg for the Cd^2+^ and Sn^2+^ ions with a contact duration of 15 min, and a dosage of 20 mg/100 mL with pH 5 for the Hg^2+^ and Pb^2+^ ions, and the study indicated that linking CNTs with ZnFe_2_O_4_ improved heavy metal ion adsorption by 25 percent [92].

#### 3.1.3. Porous Carbon Adsorbents

For the removal of heavy metal pollutants from wastewater, porous carbon compounds are regarded as valuable sorbents. These materials are typically mesoporous and macroporous, with limited micropore volume, and they facilitate the adsorption of heavy metals from the liquid phase. Because porous carbon materials are carbonaceous substances with a good degree of porosity and a large particle surface area, they have been a popular adsorbent for many years [93,94,95]. Through carbothermal reduction, Zhuang et al. (2014) developed a new porous carbon (PC)-encapsulated iron composite. The produced Fe/PC composite was tested for Cr(VI) removal in contaminated water [96], and the results were compared to commercial activate carbon (AC) and activated carbon fiber (ACF). All samples were treated to wastewater containing 5 mg/L Cr(VI), and it was discovered that the Fe/PC composite eliminated 80 percent of Cr(VI) in 10 min and 99 percent in 90 min. Under the same circumstances, AC and ACF removed only 62% and 55% of Cr(VI) ions, respectively. The results show that Fe/PC composite is a viable heavy metal adsorbent for wastewater treatment. Wu et al. (2021) reported the synthesis of hierarchical porous carbon from industrial solid waste of coal gangue and examined its performance in removing Cr(VI) ions in a separate investigation. The results indicated that the Cr(VI) adsorption capacity of hierarchical porous carbon was 320 mg/g. XPS investigations demonstrated further that hierarchical porous carbons influenced the chemisorption of Cr (VI). In order to eliminate inorganic contaminants such as Cr(VI) from wastewater, the research presented a technique for manufacturing hierarchical porous carbon from industrial waste coal gangue [97].

### 3.2. Metal Nanoparticles

Metal nanoparticles, such as silver and gold nanoparticles, have been used for years to remove heavy metal ions from wastewater. Silver, gold, and iron metal nanoparticles are the most extensively utilized adsorbent materials due to their huge surface area, outstanding optical and chemical properties, high absorptivity, and the ability to adjust their surfaces using various stabilizing and capping agents.

#### 3.2.1. Silver Nanoparticles (AgNPs)

Based on many findings, metal nanoparticles may eliminate heavy metal ions from wastewater. There are several findings in the literature of silver nanoparticles interacting with pollutants including Cd^2+^, Hg^2+^, Cr^2+^, and so on [37,98]. In terms of adsorbent performance, silver nanoparticles (AgNPs) exhibit favorable performance due to their superior physicochemical characteristics AgNPs have a high catalytic activity, biocompatible, have a good sorption potential because of their large surface area, in addition to the fact that they are easily separable and reusable [99,100]. Using a wet chemical approach, Attatsi et al. investigated how effective silver nanoparticles were in removing heavy metal ions from groundwater over a period ranging from one to fourteen days. The heavy metal ions under investigation were cobalt and lead ions. The results showed that cobalt and lead were eliminated with 24 percent and 77 percent efficiency, respectively. It is important to note that when the incubation durations were prolonged, the concentrations of metal ions decreased [101,102]. Ituen et al. disclosed the creation of silver nanoparticles from walnut husk extract. These nanoparticles were then utilized to assess how successful they were in removing heavy metal ions from petroleum effluent. The obtained findings demonstrated that the removal efficiency of Cr^2+^, Cd^2+^, and Pb^2+^ ions was 88.3%, 81.1%, and 72.6%, respectively. Furthermore, even after 4 months of storage, the walnut husk extract-produced silver nanoparticles remained stable, efficient, and thermally resistant. Overall, silver nanoparticles effectively removed heavy metals from petroleum effluent; however, the amount of heavy metal ions removed varied depending on the silver nanoparticle concentration, temperature, time, pH, and heavy metal ion dose [102]. Likewise, Negi et al. reported the effect of Cd^2+^ and Pb^2+^ ion removal from aqueous solution using freshwater algal extract-synthesized silver nanoparticles. At an adsorbent dose of 0.5 g/L, the maximum adsorption capacity for lead was 23.98 mg/g, while the maximum adsorption capacity for cadmium was 22.47 mg/g. The higher the R2 number, the better the experimental data matches the Langmuir isotherm. The kinetics studies revealed that the Pb^2+^ adhesion to silver nanoparticle surfaces was due to the electrostatic attraction, whereas Cd^2+^ adhesion to silver nanoparticles surfaces was due to physical adsorption. According to rate kinetic calculations, the adsorption of Cd^2+^ and Pb^2+^ on silver nanoparticles followed pseudo-first-order kinetics, (R2 > 0.8) and pseudo-second-order kinetics (R2 > 0.9), respectively. This was determined by comparing the values of the rate constants. Finally, the findings suggest that silver nanoparticles might be applied as adsorbents in wastewater treatment to eliminate heavy metal ions in an effective manner [103].

#### 3.2.2. Gold Nanoparticles (AuNPs)

Gold nanoparticles are commonly employed in the treatment of wastewater to detect and remove heavy metal ions (Table 1). Gold nanoparticles are very effective at removing heavy metal ions and have shown high selectivity for a range of metal ions [98]. Given the large surface area of gold nanoparticles and their superior optical characteristics, as well as their greater molar absorptivity, they stand out when compared to other metallic nanoparticles. As a result, they are frequently utilized for colorimetric biosensing and removal of a wide range of targets. Additionally, the surfaces of gold nanoparticles can be modified with various capping and stabilizing agents, allowing them to be used in a variety of applications [104]. The development of biosensing technologies, which has been aided by the advancement of bio nanotechnology, has opened new options for the easy detection of heavy metals. Colorimetric biosensing using AuNPs functionalized with DNA provides great sensitivity towards targets, as well as ease, user friendliness, and the elimination of equipment, all of which are desired for field analysis. It is widely believed that when DNA binds with heavy metal species, their confirmation and coupling status change, which significantly modify the interfacial contacts of the DNA–AuNPs, causing the assembly/disassembly of the DNA–AuNPs to represent the target heavy metal species [105]. Tan et al. investigated the impact of binding aptamer strands of different lengths on AuNPs for colorimetric sensing of Hg^2+^. To begin, the 15T bases were developed with the purpose of detecting Hg^2+^ by the coordination of T–Hg^2+^–T, which resulted in a detection limit of 9.6 × 10^−9^. Simply increasing the number of A bases just on one or either sides of the 15-mer sequences was all that was required to lengthen or shorten the 15-mer DNA sequence to generate the 25-mer and 59-mer sequences, respectively. The findings showed that decreasing the length of the DNA chain did not improve the detection sensitivity. Therefore, the acquired sensitivity towards the Hg^2+^ increased in the order of 15-mer < 25-mer < 59-mer aptamer. The apparent increase was attributed to the following factor: in the presence of Hg^2+^, T–Hg^2+^–T coordination ended up causing T base sequences to fall off the surfaces of the AuNPs, while additional A base sequences remained adsorbed on the surface of the AuNPs, which resulted in morphologically evident variations in grown AuNPs, allowing visible solution color changes [106]. Ruíz-Baltazar et al. reported diatoms–AuNPs (Dtm–AuNPs) composites for heavy metal ion adsorption. The *Piper auritum* extract was employed as a reducing agent for AuNPs, and the composite was produced by ultrasonification. The ability of Mn^2+^ adsorption was tested using Dtm–AuNPs composites through varying the concentration of the composite from 1.0 mg/L^−1^ to 20 g.L^−1^. The adsorption procedure, which was aided by sonication, attained 98% adsorption effectiveness in 240 min. Furthermore, linear and non-linear pseudo-second-order models agreed well with the experimental data. Overall, the research demonstrated considerable Mn^2+^ adsorption using the Dtm–AuNPs composite, which is a novel, low-cost, and environmentally acceptable material for Mn^2+^ adsorption [107].

#### 3.2.3. Iron Nanoparticles

Iron nanoparticles have been extensively employed as adsorbents to eliminate heavy metals from wastewater due to their strong redox and adsorption abilities. It has been shown that the use of iron nanoparticles may effectively minimize the potential leachability of toxic substances via the process of in situ immobilization. In the beginning, materials made of iron were used in reactive subsurface permeability shields in order to extract chlorinated chemicals from groundwater. The development of nanotechnology led to the substitution of conventional ferromagnetic materials and large-scale iron particles with iron nanoparticles that have great activity. The findings of the experiments demonstrated that the reaction rate constant of iron nanoparticles was higher than that of conventional iron powder. Accordingly, it has been shown that iron nanoparticles may perform the functions of both reductants and catalysts in order to eliminate heavy metal pollutants [108]. Recently, Bhattacharjee et al. (2021) reported the novel green approach for the synthesis of iron sulfide nanoparticles and studied their efficacy in removing hexavalent chromium. The results revealed that 50% of chromium was removed within 5 min of contact time, and the total removal of chromium was observed under the condition of 4.5 mg/L chromium by iron sulfide nanoparticles at a dosage of 0.5 g/L at pH 7 and contact time of 120 min [109]. Saleh et al. (2021) reported the manufacture of zerovalent iron nanoparticles with *Verbascum thapus* leaf extracts serving as reducing and stabilizing agents. When the generated nanoparticles were investigated for their capacity to reduce Cr(VI) ions, it was discovered that at a dosage of 1 g/L with a contact time of 30 min, they were able to completely eliminate the Cr(VI) [110]. Hence, the above studies suggest that the iron nanoparticles can be effectively employed in wastewater treatment.

### 3.3. Metal Oxide Nanoparticles

Nanoparticles of metal oxide have a high capacity for heavy metal ion adsorption. Nanoparticles of metal oxides such as CuO, TiO_2_, MgO, ZnO, and CeO_2_ are extremely promising for the removal of heavy metal pollution from water bodies due to their large surface area and their ability to remove heavy metal ions, even at low concentrations.

#### 3.3.1. Copper Oxide (CuO) Nanoparticles

Copper is a naturally occurring and ubiquitous element in the environment. Its concentration in the Earth’s mantle is approximately 60 g/ton. Copper oxide nanoparticles, also known as CuO NPs, have been shown to be good adsorbent materials due to their low cost, ease of production, smaller size, greater surface area, abundant supply of their precursor material, and absence of toxicity. In wastewater treatment, CuO NPs are widely employed as an adsorbent to extract heavy metals, dyes, medicinal compounds, and other contaminants (Table 1). The highest recorded fluoride adsorption capacity of CuO NPs was 3152 mg/g [111]. This is rather impressive, since it shows that the adsorbent can extract fluoride from the aqueous medium in excess of three times its weight. In addition, higher adsorption was observed for mercury, malachite green, Congo red, and methylene blue [111]. In a study, the adsorption efficacy of CuO nanoparticles for removing heavy metal ions such as Cd^2+^ and Fe^3+^ was evaluated by altering the physical parameters, including metal ions and adsorbent concentration, equilibrium contact duration, and aqueous solution pH. According to the findings, the adsorption capacity rose as the pH of the aqueous solution increased, with pH 6 being the optimal pH for adsorption of both metals. The kinetic analyses demonstrated that the adsorption of both metal ions on CuO NPs exhibited a pseudo-second-order behavior, and the equilibrium adsorption data suggested that the Langmuir isotherm model best represented Cd^2+^, while the Freundlich isotherm model best represented Fe^3+^. According to the findings, CuO NPs have the potential to act as an adsorbent in the process of removing heavy metal ions from the aqueous phase [112]. Hassan et al. similarly detailed the wet chemical synthesis of CuO nanoparticles and assessed their efficacy in removing lead from aqueous solution. Using CuO NPs, lead was removed from its aqueous solution by adsorption with an 84 percent removal effectiveness, according to the findings. In a separate work, Mahmoud et al. demonstrated an eco-friendly green synthesis approach for the creation of CuO nanoparticles using orange peel and mint leaf extracts. The performance of the produced nanoparticles in removing Pb^2+^, Ni^2+^, and Cd^2+^ ions was investigated. For Pb^2+^, Ni^2+^, and Cd^2+^, the optimum absorption capacity of each CuO NP was estimated as 88, 54, and 15 mg.g^−1^, respectively. Furthermore, Freundlich and pseudo-second-order models provided the best interpretation of the experimental results, and adsorption equilibrium was achieved in 1 h. These results suggest that CuO NPs could be a suitable nanoadsorbent for detoxifying water polluted with heavy metals [113].

#### 3.3.2. Titanium Dioxide (TiO_2_) Nanoparticles

TiO_2_, among other metal oxide nanoparticles, have found widespread application for their ability to eliminate heavy metal ions. TiO_2_ nanoparticles are more promising than other metal oxide nanoparticles owing to their distinct physical–chemical characteristics (Table 1), which include a large surface area, non-toxic behavior, and photocatalytic properties. Photocatalysis is one of the possible approaches for reducing harmful toxins, and TiO_2_ has been intensively researched as a photocatalytic material for polluted environmental remediation. The photocatalytic effectiveness of TiO_2_ nanoparticles in removing Cd^2+^ and Pb^2+^ was investigated by Rahimi et al. The findings revealed that pH had a significant impact on Cd^2+^ and Pb^2+^ adsorption rates. It was also shown that raising the TiO_2_ concentration and pH values enhanced metal removal efficiency. Similar to their unmodified counterparts, photocatalytic removal of Zn and Pb ions from an aqueous solution utilizing vanadium-doped TiO_2_ nanoparticles resulted in greater metal ion removal efficiency for vanadium-doped TiO_2_ nanoparticles. Vanadium-doped TiO_2_ nanoparticles exhibited increased metal ion removal efficiencies from 7 to 11 mg.g^−1^ for Zn and 17 to 26 mg.g^−1^ for Pb ions, respectively [114]. TiO_2_ nanoparticles are increasingly being employed in wastewater treatment due to their remarkable adsorption capabilities. Herrera–Barros et al. reported that Ni^2+^ could be removed through an adsorption process utilizing lemon peel biomass enhanced with TiO_2_ nanoparticles. To investigate the adsorption effectiveness of Ni^2+^ ions, pH and particle size of biomass were varied. The findings demonstrate that at pH 6, the Ni^2+^ ion adsorption rate of 78% was observed, but no significant changes were observed for biomass particle size. Furthermore, the overall findings show that grafting TiO_2_ nanoparticles onto lemon peel biomass boosted the adsorption efficiency of Ni^2+^ ions, implying that biomass treated with metal oxide nanoparticles has the ability to remove heavy metal ions [115]. Moreover, cellulose acetate membrane modified with amine-impregnated TiO_2_ nanoparticles exhibited greater efficiency in removing chromium (VI) ions from aqueous solution. However, the metal ion concentration, amine type, and solution pH all had an influence on removal efficiency [116,117]. The major disadvantages of using TiO_2_ nanoparticles are its complicated fabrication process and difficulty in separation when used in slurry suspension [4].

#### 3.3.3. Magnesium Oxide (MgO) Nanoparticles

As adsorbents for heavy metal ions, magnesium oxide offers several benefits, which include an exceptional adsorption capacity, low cost, low toxicity, good availability, and an eco-friendly behavior [114]. Metal ion removal efficiency is higher in nanosized MgO than in commercially available bulk MgO (Table 1). MgO nanoparticles had a removal capability of 96% of Cu^2+^ ions from a copper chloride solution (10 ppm), while commercially available MgO had a removal capacity of 15% [118]. Cai et al. observed the removal of toxic metal ions as well as bacterial inactivation using MgO nanoparticles. MgO nanoparticles fabricated using the sol–gel method were employed to eliminate both Cd^2+^ ions and *Escherichia coli* bacteria. The findings showed that 100 mg/L of MgO nanoparticles eliminated 50 mg/L Cd^2+^ ions while also inactivating 7-log bacterial cells. Furthermore, MgO nanoparticles were found to have increased bacterial inactivation in the presence of Cd^2+^ ions. This might be attributable to MgO’s direct contact with the bacterial cell membrane, which caused membrane degradation and the entry of Cd^2+^ ions into the cell body, resulting in intercellular component leakage. This research suggests that MgO nanoparticles might be a suitable material for wastewater treatment polluted by heavy metal ions and bacteria [119]. MgO nanocomposites, in addition to nanoparticles, have been extensively studied for their high adsorption properties against heavy metal ions. Habiby et al. demonstrated phosphate ion removal using MgO/Fe_3_O_4_ nanocomposites. The adsorbent was found to have superparamagnetic characteristics with a surface area of 75 m^2^/g. According to the adsorption investigation, the maximum phosphate adsorption efficiency of 98.9% was attained after 20 min of contact time at 25 °C, pH 9, with a dosage of 5 g/L MgO/Fe_3_O_4_ nanocomposite and a metal ion concentration of 1 mg/L. This was accomplished using a concentration of metal ions and a dosage of 5 g/L MgO/Fe_3_O_4_ nanocomposite. The adsorption process was exothermic and spontaneous, as evidenced by the thermodynamic characteristics, and the pseudo-second-order kinetic model suited the experimental results well [120]. Abuhatab et al. used MgO- and NiO-embedded silica-based nanocomposites to explore the adsorptive elimination of Cr^3+^, Cu^2+^, and Zn^2+^ ions. The maximal absorption values were found to be 41.36, 13.76, and 7.23 ions/nm^2^ for Cr^3+^, Cu^2+^, and Zn^2+^, respectively. Furthermore, when the pH of the metal ions was elevated from 7 to 11, the adsorption rate increased. These findings suggest the MgO- and NiO-embedded silica-based nanocomposite may be a candidate material for eliminating heavy metal pollutants from water bodies [121].

#### 3.3.4. Zinc Oxide (ZnO) Nanoparticles

ZnO nanostructures possess distinguished physicochemical and biological properties that make them distinctive from ZnO in bulk form (Table 1). The key factors that determine the physicochemical characteristics of ZnO nanostructures are their structure, particle size, surface features, and crystallinity. Because of its remarkable adsorption capacity towards heavy metal ions, ZnO has gained attention as a nanoadsorbent in recent years. ZnO nanoparticles are effective adsorbent materials because of their porous nanostructures and large surface areas [122]. The performance of ZnO nanoparticles in removing Cr^6+^, Ag+, Cu^2+^, Mn^2+^, Pb^2+^, Ni^2+^, and Cd^2+^ was investigated by Le et al. It was shown that the ZnO nanoparticles generated via solid precipitation had a rod-like structure, with an average length of 497 nm and a diameter of 75 nm, respectively. According to the findings, ZnO nanoparticles were very effective in removing Cu^2+^, Ag^+^, and Pb^2+^ ions, with an efficiency of removal that was more than 85%. However, the removal effectiveness of Cr^6+^, Mn^2+^, Cd^2+^, and Ni^2+^ ions was shown to be less than 15%. In addition, it has been stated that the process for heavy metal ion removal was found to be reduction for Cu^2+^, Ag^+^, and Cr^6+^ ions; oxidation for Pb^2+^ and Mn^2+^ ions; and adsorption under UV irradiation for Cd^2+^ and Ni^2+^ ions. All of these pathways were discovered to be responsible for heavy metal ion separation or removal [123]. Gu et al. looked at the adsorption of heavy metal ions from dental effluent in another study. After using the hydrothermal method to produce ZnO nanoparticles with a specific surface area of 26.777 m^2^/g, these nanoparticles were put through a series of tests to see how well they removed Cr^3+^ metal ions. In adsorption experiments, ZnO nanoparticles were shown to have a greater affinity for Cr^3+^ ions, with a maximum adsorption of 88.5 mg/g for 1 g of adsorbent in 1 L of water. Moreover, the pseudo-second-order and Langmuir isotherm models best describe the adsorption experiment data. As a direct consequence of this, the ZnO nanoparticles that were generated have the potential to be useful material for the filtration of dental effluent [124].

#### 3.3.5. Cerium Oxide (CeO_2_) Nanoparticles

Cerium oxide is a non-toxic rare-earth metal oxide that has found widespread application in a variety of fields, including photocatalysis, UV blocking, sensor technology, and the treatment of wastewater (Table 1). CeO_2_ nanoparticles have also been studied in terms of specific surface area, indicating that nanoparticles are potent adsorbents with size-dependent properties [4]. Contreras et al. looked at how pH affected the adsorption process of heavy metal ions such as chromium (VI), lead (II), and cadmium (II). Utilizing factorial experimentation, an empirical equation that describes the sorption capacities of CeO_2_ nanoparticles as a function of the starting concentrations of both metal ions and nanoparticles as well as pH was created. The study discovered that CeO_2_ nanoparticles had the highest absorption for Cd^2+^ at 93 mg/g, Pb^2+^ at 128 mg/g, and Cr^6+^ at 34.4 mg/g. Furthermore, the findings showed that the sorption capacity of CeO_2_ nanoparticles was approximately equivalent in both single and multiple systems, and that the initial metal ion and nanoparticle concentrations were significantly predictive of all three metals’ sorption capacities. The sorption capacity of cadmium and chromium was shown to be affected by pH but not lead. This research paves the way for the novel use of CeO_2_ nanoparticles in the capacity of an adsorbent for complex waterways that are contaminated with heavy metals. In situ growth and self-assembly were employed to assess the capacity of CeO_2_ nanoparticles to remove Cd^2+^, Pb^2+^, and Ce^6+^ from an aqueous solution when combined with reduced graphene oxide. The in situ-grown CeO2/rGO nanocomposite had the highest lead adsorption rate (95.75 mg/g), whereas the self-assembled nanomaterial had the highest cadmium adsorption rate (31.26 mg/g), according to the data. The kinetic experiments found that the Langmuir model matched the lead removal best, whereas the Freundlich and Langmuir models fit the cadmium removal best. Although CeO_2_/rGO showed strong Cd^2+^ and Pb^2+^ adsorption capacities, only a minor concentration of Cr^6+^ was removed [125]. According to the results, the CeO_2_/rGO nanocomposite produced via in situ growth could be a potential material for wastewater treatment.

#### 3.3.6. Other Metal Oxide Nanoparticles

The application of iron oxide nanoparticles for the elimination of toxic metals from wastewater is more attractive due to their unique properties, such as their magnetic properties, huge surface area, and tiny size. Because iron oxide nanoparticles are magnetic, it is simple to extract adsorbent materials from the solution for easy reusability [126]. Yang et al. (2021) recently fabricated a magnetically separable and recyclable Fe_3_O_4_/polydopamine-grafted L-cysteine (Fe_3_O_4_/PDA/L-Cys) core shell composite and investigated its efficiency in removing Pb^2+^ ions from aqueous solution. At pH ranges between 2.8 and 6.0, the Pb^2+^ removal effectiveness of Fe_3_O_4_/PDA/L-Cys was evaluated. The research demonstrated that adsorption increased as pH rose. At a concentration of 1.5 g/L, the maximum Pb^2+^ ion adsorption was reported at a pH of 6. The kinetic analyses revealed that the adsorption conformed well to the pseudo-second-order and that the Fe_3_O_4_/PDA/L-Cys removal was spontaneous and endothermic [127]. In another study, Jian et al. (2022) investigated the effect of Ln–Mn bimetal oxide nanofibers on removing fluoride ions. The findings showed that the Ln–Mn bimetal oxide nanofibers performed well in terms of defluoridation. According to the kinetic analyses, the adsorption reached equilibrium after 90 min, and the adsorption was best described by pseudo second-order kinetics. Furthermore, the Ln–Mn bimetal oxide nanofibers were found to be recyclable [128]. Likewise, Jian et al. (2022) published the fabrication of magnetic La_2_O_3_/CeO_2_/Fe_3_O_4_ nanofibers and studied their efficiency in removing fluoride ions. The produced La_2_O_3_/CeO_2_/Fe_3_O_4_ nanofibers demonstrated good defluoridation, with a maximum absorption of 229 mg/g recorded. The findings of thermodynamic analysis demonstrated that the defluoridation process was spontaneous and endothermic. Kinetic investigations demonstrated that the adsorption mechanism was well-described by a pseudo-second-order model. The prepared La_2_O_3_/CeO_2_/Fe_3_O_4_ nanofibers were good at regenerating, which suggests that La_2_O_3_/CeO_2_/Fe_3_O_4_ nanofibers are stable and promising options for defluoridating wastewater [129].

#### 3.3.7. Biomass-Derived Nanomaterials

Materials made from biomass are widely available, cheap, and have very little to no economic value. Despite all of these factors, they are more prevalent and pose several handling and disposal challenges [130]. Multipurpose carbon-based nanomaterials have been prepared utilizing waste byproducts as the primary inputs, drawing inspiration from the transformation of waste biomass into high-value products. As a top priority for study in the area of nanotechnology, carbon nanotubes (CNTs) stand out above other prevalent carbon-based nanomaterials. Researchers have shown that modified CNTs had a higher adsorption capacity than raw CNTs, despite the fact that both are appropriate as heavy metal ion adsorbents. Therefore, the inclusion of functional groups (such as hydroxyl, carboxyl, and carbonyl groups, mostly carboxylic) has produced many adsorption sites, therefore boosting the adsorption capacity of CNTs [131]. Sachan et al. (2021) prepared biomass-derived silica nanoparticles using *Saccharum ravannae*, *Saccharum officinarum*, and *Oryza sativa* leaf extracts. The ability of biomass-derived silica nanoparticles to remove Cu^2+^ and Pb^2+^ from wastewater was investigated further. In addition, the Freundlich isotherm model and pseudo-second-order kinetics were employed to characterize the adsorption process [132]. Chen et al. (2017) synthesized mesoporous carbon-embedded iron carbide nanocomposites from natural biomass for the efficient removal of heavy metals from wastewater. The cotton fabric biomass was employed as a carbon precursor, and mesopores were created via a graphitization reaction catalyzed by iron. Maximum heavy metal adsorption was found to occur in the following order: Cr(VI) > Pb(II) > Cu(II) > Ni(II) > Zn (II). Additionally, it was noticed that the rate of adsorption decreased as the regeneration cycles increased. Previous research indicated that materials generated from biomass are good at eliminating heavy metals from wastewater [133]. Nanomaterials for heavy metal removal are shown in Table 2.

## 4. Role of Adsorptive Nanocomposite Membranes in Heavy Metal Removal

A wide range of processes, such as reverse osmosis, coagulation, membrane filtration, chemical reduction, electrolytic removal, ion exchange, flocculation, precipitation, phytoremediation, photocatalytic degradation, electrodialysis, and adsorption, have been utilized in the treatment of wastewater [153]. Due to its scalability, high efficacy, ease of operation, low cost, and the ability of adsorbents to be regenerated, adsorption has been shown to be one of the most successful approaches. Utilizing a wide array of adsorbents has enabled the removal of several pollutants from wastewater, particularly heavy metals and metalloids [154].

In the 1980s, membrane adsorbent technology was created by combining membranes with adsorbents that bind molecules through physical or chemical interactions [155]. Broadly, chemical adsorption is advantageous since the contacts are stronger, allowing for more adsorption capacity [156]. There are several methods for creating adsorptive membranes. Blending of two or more homopolymers, the use of copolymer membranes, the grafting of adsorptive molecules onto the polymer membrane, and the dispersion of adsorbents in screens (mixed matrix membranes) are common fabrication techniques [157]. In addition, chemical post-modification of (polymeric) membranes is a potent technique for creating adsorptive membranes. Previous research has shown that membranes modified with nitrogen-containing groups, such as amines and imines, had promising anion adsorption and cation adsorption properties [158,159]. However, post-modification processes for ion-absorbing membranes are often undertaken on fiber mat or microfiltration membranes.

Sarah Glass et al. developed the post-modification of polyacrylonitrile (PAN) membranes with primary, secondary, tertiary, and quaternary amines using different modification reactions. In comparison to other membrane materials, PAN is chemically easier to modify, since it is carrying a nitrile group. Several modification reactions of nitrile-carrying substances such as PAN are known. Nevertheless, these modification reactions were usually not applied on PAN ultrafiltration (UF) membranes, which require heterogeneous reactions in order to preserve the membrane structure [160].

Adsorbents such as carbon-based nanomaterials, zeolites, polymers, agro-waste, ceramics, metal oxides, composites, and hybrid materials have been widely used for the adsorptive removal of a wide range of pollutants from contaminated water. This procedure is referred to as adsorption. Adsorption refers to the physical and chemical interaction that happens between an adsorbate and an adsorbent. The adsorption process is affected by a variety of characteristics, including but not limited to temperature, medium pH, adsorbent–adsorbate interaction forces, concentration, the presence of foreign components, and others [154,161].

The adsorption process is based on mass transfer in which a material accumulates at the interfaces of two phases, such as the contact between liquid and solid, liquid and liquid, or gas and solid [34]. This process is categorized into three categories: chemisorption, physisorption, and ion exchange [161].

Some nanoparticles can selectively remove a large variety of specific substances from polluted water [162]. When compared to a pristine membrane, adding nanoparticles to the membrane improves pure water permeability [163]. Nano-sized adsorbents may be utilized in adsorptive nanocomposite polymeric membranes for several reasons. These include the following: fluctuations in surface charge and large surface areas are two of the properties that have been demonstrated. These qualities are responsible for the material’s high adsorption capability and selectivity, as well as its high porosity and significant affinity for particular heavy metal species. In addition, the insertion of nanoparticles into polymeric membranes does have the potential to alter the physicochemical properties of the original membrane, which might result in an increase in the membrane’s water permeability and mechanical strength [25].

### 4.1. Mechanical Strength

**Figure 3:**
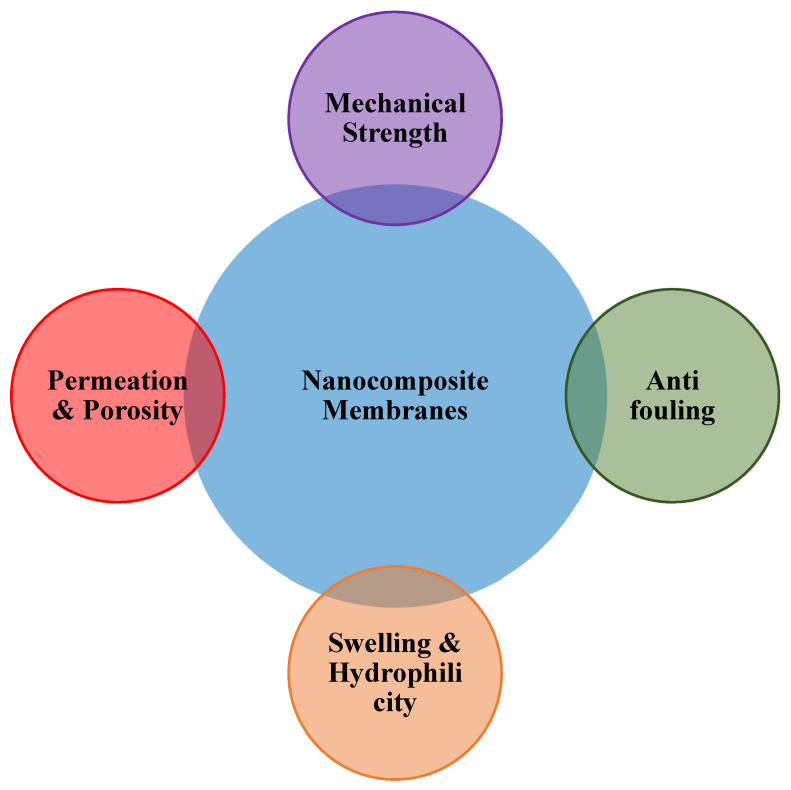
Important properties in the development of different nanocomposite membranes.

It has been found that the mechanical strength of adsorbent nanocomposite membranes is an outward sign of their durability (Figure 3). Researchers have utilized elongation at break, tensile strength, and Young’s modulus to better comprehend the capacity of adsorptive membranes to survive elastic deformation and stress under demanding working conditions. In general, increasing the nanoadsorbent concentration enhanced the mechanical strength of the adsorptive membranes; however, when the nanoadsorbent critical concentration was surpassed, the mechanical strength of the adsorbent membranes dramatically dropped. Adsorptive membranes with optimal nanoadsorbent loading enhance the mechanical strength of the structure by enhancing the contact between nanoadsorbents and the polymer matrix, forming a thicker skin layer and suppressing macro voids.

According to Mukherjee, the mechanical properties of the polysulfone/graphene oxide nanocomposite membrane rose as the graphene oxide concentration rose from 0 to 0.1 percent by weight in the polysulfone membrane. The formation of a connection between two polysulfone polymer chains by graphene oxide nanoparticles resulted in greater contact between the nanocomposite membranes. However, when the graphene oxide nanoparticle loading climbed to 0.5% weight, the membrane’s mechanical strength decreased before the pore density in the membrane structure increased [164].

Amir Razmjou et al. found that with minor differences (TiO_2_/Al_2_O_3_/SiO_2_ PVDF nanocomposite membrane) in tensile strength between control and nanocomposite membranes, the elongation at break for composite membranes decreased dramatically [165].

### 4.2. Permeability

The water permeability (Figure 3) of adsorptive membranes is determined by their hydrophilicity, pore size, skin thickness (swelling), porosity, and surface roughness. The majority of the increase in water permeability in adsorptive membranes is due to increasing pore size and porosity. In one of the research studies, the introduction of graphene oxide nanoparticles into a polysulfone membrane produced larger holes in the membrane’s middle cross-sections [164]. In addition, the use of nanoparticles enhanced water’s hydrophilicity and membrane structure. Thus, there was little interaction between polymer and solvent molecules, which permitted solvent molecules to migrate easily from the polymer matrix into the coagulation bath [166]. In addition, it was found that the mean pore size of the membrane decreased due to the higher viscosity of the casting solution caused by the incorporation of nanoparticles into the membrane matrix. Consequently, the rate of exchange of solvent and other particles slowed during the phase inversion procedure [167].

Generally, an increase in membrane surface roughness results in increased water permeability because of the greater incorporation of hydrophilic nanoparticles. This was corroborated by Abdullah et al. [168], who reported that increasing the loading of hydrophilic hydrous ferric oxide nanoparticles in a mixed matrix membrane led to a rise in the membrane porosity, hydrophilicity, water permeability, and roughness. By modifying the surface area and water permeability of the membranes, they were able to draw more water molecules to the surface [168].

Moreover, the swelling properties were directly related to the membrane’s water content. Pure water flow rose by 3.2 percent when polyaniline concentration was raised from 5 to 10% in the membrane [169]. Likewise, pristine chitosan membrane swelled from 33.91 to 37.70 percent when 1.25 percent weight zeolite nanoparticle was added [170]. When comparing the hydrophilicity of nanomodified membranes to clean membranes, the hydrophilicity of the nanocomposite membrane improved in contrast to a clean membrane due to the migration of nanoparticles to the upper surface of the nanocomposite membrane, resulting in enhanced permeability and flow rate. The water contact angle affects the hydrophilicity of a membrane. The greater the angle of water contact, the greater the hydrophilicity, resulting in enhanced water flow and separation qualities [163]. Findings of membrane contact angle experiments revealed that adding TiO_2_ to the membrane resulted in reduced contact angles, indicating enhanced hydrophilic characteristics [165].

### 4.3. Surface Charge Alteration

One can accomplish higher than 90% heavy metal removal during waste water treatment by changing the surface charge of nanocomposite material membranes [171]. Surface modification (Figure 3) with different functional groups could result in the surface charge of the membranes (carbon nanotubes, graphene oxide, etc.), which would immensely increase the adsorption capacities [172,173].

When carbon nanotubes were functionalized with boronic acid, the surface charge decreased to −37 mV from −7 mV, resulting in an increase in tetrahedral anions, which increased metal adsorption, namely, Ni (II), Cr (VI), and Cu (II) by 205.7 mg/g, 175.67 mg/g, and 183.38 mg/g respectively, at a pH of 7, 2, and 3, respectively [174].

Graphene oxides, on the other hand, are utilized to remove heavy metals by increasing the adsorption behavior, since they can be readily controlled by surface functionalization using various functional groups [175]. The elimination of Hg (II) from a 1 ppm metallic solution increased from 75% to 89% when oxygen-rich groups were added to the GO/Fe–Mn composite. Using a model of surface complexation, Fe–Mn oxides on the surface of graphene oxide attracted Hg (II) and made mercury removal from a metallic solution simpler. This was accomplished by exchanging ligands between the surface –OH groups of GO/Fe–Mn and Hg(OH)_2_ [176].

In another research study, nickel iron oxide was added to polysulfone nanocomposite membranes, at which point the membrane’s zetapotential plummeted from 8.8 mV to −49 mV. In order to attain an equilibrium, hydrophilic NFO nanoparticles were distributed over the polymer–water interface during phase inversion. This resulted in negative surface charges of the adsorbent membranes [177].

### 4.4. Mechanisms of Heavy Metal Ions via Adsorptive Nanocomposite Membranes

Through ion exchange or surface complexation, adsorptive membranes can be changed to incorporate reactive functional groups such as –COOH, –NH_2_, and –SO_3_H groups. These functional groups are needed for the attachment of metal ions; hence, they are eliminated upon contacting the membrane surface, regardless of whether the pore diameters are larger than the metal ion size. To properly define the removal mechanism of heavy metals via adsorptive membranes, it is essential to recognize that their surfaces are charged by ionization, and that the adsorbent may include charged ionic functional groups. For the removal of arsenic from water, Rowley et al. created polyethersulfone (PES) nanocomposite membranes utilizing surface-modified Fe_3_O_4_ nanoparticles (NPs). The PES membranes produced with A–Fe_3_O_4_ NPs exhibited effective adsorption capabilities with low concentrations of NPs (1 wt.%, 2 wt.%, and 3 wt.%), indicating a promising impact for industrial applications in the water treatment sectors. PES membranes with 3 wt.% A–Fe_3_O_4_ NPs had the greatest rejection rate of 76% and the highest observed arsenic equilibrium adsorption capacity of 14.6 mg/g [178].

## 5. Fabrication of Nanocomposite Membranes

In this review, we discuss different techniques that are employed to fabricate the different kinds of nanocomposite membranes. Firstly, the nanocomposite membranes are differentiated into four types: conventional nanocomposites, thin-film nanocomposites, thin-film composites with nanocomposite substrates, and surface-located nanocomposites and mixed matrix nanocomposite membranes (MMNMs) [179]. MMNMs are the emerging advanced membranes for water treatment, including nanoparticles distributed in a polymer matrix [180]. For MMNMs, nanomaterials can be incorporated into polymers to provide unique functionality to membranes, such as antibacterial and photocatalytic properties. Substantial interactions between the interface of nanoparticles and the polymer matrix that surrounds them have the potential to increase the physical properties of the membrane, such as its strength and modulus [181].

Usually there are three approaches for the first step (homogenous solution synthesis of nanocomposite membranes). The polymer is added to the solvent initially, and then the nanofiller is added to the mixture in one method. In another, the nanofiller is added to solvent and the polymer to that mixture. In a third method, the polymer and nanomaterials are dissolved in solvent separately and combined to yield a homogenous mixture [182]. Of these different approaches, last two are the more preferred methods for inorganic particle dispersion.

Then the different fabrication techniques come into play in producing nanocomposite membranes.

### 5.1. Phase Inversion

**Figure 4:**
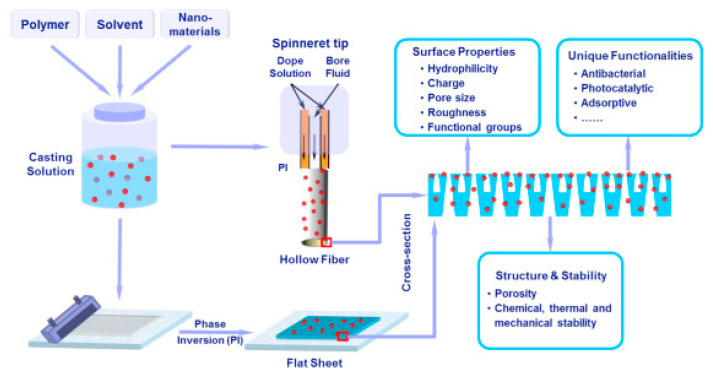
Nanocomposite polymer-matrix membranes for water purification [179].

Phase inversion is the controlled de-mixing (liquid–liquid de-mixing) of homogeneous polymer solutions into two solid and liquid phases [183]. In the process of demixing, the polymer-rich phase becomes destabilized as a result of an increase in polymer concentration and a decrease in polymer solubility. The polymer begins to harden and become insoluble as a result. The most prevalent forms of polymer membranes formed by phase inversion (Figure 4) are flat sheets and hollow fibers [179].

Four types of nanofillers employed in the phase inversion procedure are as follows: (1) organic material; (2) inorganic material; (3) hybrid material containing two or more types of material; and (4) biomaterial. Hydrophilicity, permeability, porosity, pore interconnectivity, and antifouling abilities are all improved by utilizing diverse combinations of polymers and nanofillers.

Polyanaline nanofibers were utilized as a nanofiller in the manufacture of nanocomposite membranes, increasing attributes such as membrane strength as well as hydrophilicity and pore size, which enhanced permeability through the polymer [184,185].

In one study, the combination of inorganic metals such as silver with polysulfone increased the membrane’s pore size, hydrophilicity, and antibacterial characteristics [186].

Using a phase inversion method, specific proportions of polyethersulfone and polyaniline (PANI)/Fe_3_O_4_ nanoparticles were allowed to dissolve in N,N-dimethylacetamide to create a nanocomposite membrane (400 rpm for 24 h). Polyaniline (PANI)/Fe_3_O_4_ nanoparticles were initially dispersed in N,N-dimethylacetamide using ultrasonication before being added to a polyethersulfone solution due to their magnetic nature [187].

The instantaneous phase inversion between the nonsolvent and solvent in the coagulation bath results in the creation of a permeable membrane with macrovoid gaps, a thin top layer, and finger-like cavities. This is due to the vast quantities of heavy metal ions that flow through the membrane. As per the results of a number of research studies, this technique increases the number and size of pores, which consequently enhances the diffusion rate of nonsolvents [188,189].

### 5.2. Stretching

The vast majority of microporous membranes that are used in microfiltration and ultrafiltration are created by the processes of solution casting (or an extraction method), dry stretching, and particle stretching [190,191]. In order to extract a plasticizer, first a polymeric feedstock and a processing plasticizer must be combined, then the mixture must be extruded, and lastly the plasticizer must be extracted.

A porous membrane is produced by a variety of procedures. In the first phase of the process, a precursor film with a row-nucleated lamellar structure is produced by shear- and elongation-induced crystallization of the polymer. This polymer is chosen for its high molecular weight and even distribution of molecular weight. The second stage involves annealing the precursor film at temperatures close to the melting point of the resin. This will assist in eliminating crystalline phase defects and increasing lamellae thickness. Finally, the pores are extended at low and high temperatures in order to develop and expand them suitably [192].

In one study, nanostructured stretched polytetrafluoroethylene (PTFE)–sulfonated polystyrene (PS) ion-exchange membranes were investigated for their suitable physical and chemical characteristics as well as their efficiency. To implant the PS in the stretched PTFE film, high-temperature polymerization of styrene, which was absorbed at 90 degrees Celsius from a solution consisting of styrene, divinylbenzene, and toluene, was utilized. According to the findings of the researchers, the membranes that were produced had a permeability and diffusion coefficient for methanol 1.5 times lower than those for water [193].

In addition, the initial stage in the creation of high-density polyethylene (HDPE) hollow fiber membranes was conducted to stretch the precursors of melt-spun hollow fibers. Consequently, technologies for producing slit-shaped holes by cold and hot stretching have been developed effectively. It was observed that the take-up rate affected both the microporous structure and the crystalline structure of the precursors of the stretched membrane [194].

### 5.3. Electrospinning

Electrospinning is a unique fabrication method and a straightforward nanofiber production technology that may be used to generate nanofibrous nonwovens. It is a basic and simple process for producing nanofibers with the potential for cost-effective development on laboratory, pilot, and industrial scales. Moreover, electrospun nanofibrous materials offer distinct benefits when used as membranes due to their specific properties, which include small fiber diameters, high porosity, large specific surface area, flexibility in surface functionality, excellent pore size adjustability, and pore interconnectivity [195]. These attributes contribute to the beneficial properties of electrospun nanofibrous membranes (ENM), such as low operating cost, minimal pressure drop, and high permeability.

Electrospinning is gaining popularity as a versatile and trustworthy technology for producing nanofibers for application in porous membrane production, such as water filtration and desalination. Electrospun membranes were used to separate polystyrene particles of 1, 5, and 10 m diameters. Electrospun membranes successfully removed more than 90 percent of the microparticles from solution. This study sets the way for future research on the use of nanofibers as screens in separation techniques for water pre-treatment prior to reverse osmosis or as pre-filters to reduce fouling and pollution before ultra or nanofiltration [196].

In recent years, significant advancements have been achieved in relation to this topic, and the technology in question has been used in a diverse array of applications. To better regulate nanofiber shape, structure, surface functionality, and assembly techniques, researchers have concentrated on either better understanding of the fundamental principles of electrospinning or identifying optimum electrospinning settings for diverse polymers and biopolymers [197,198,199].

**Figure 5:**
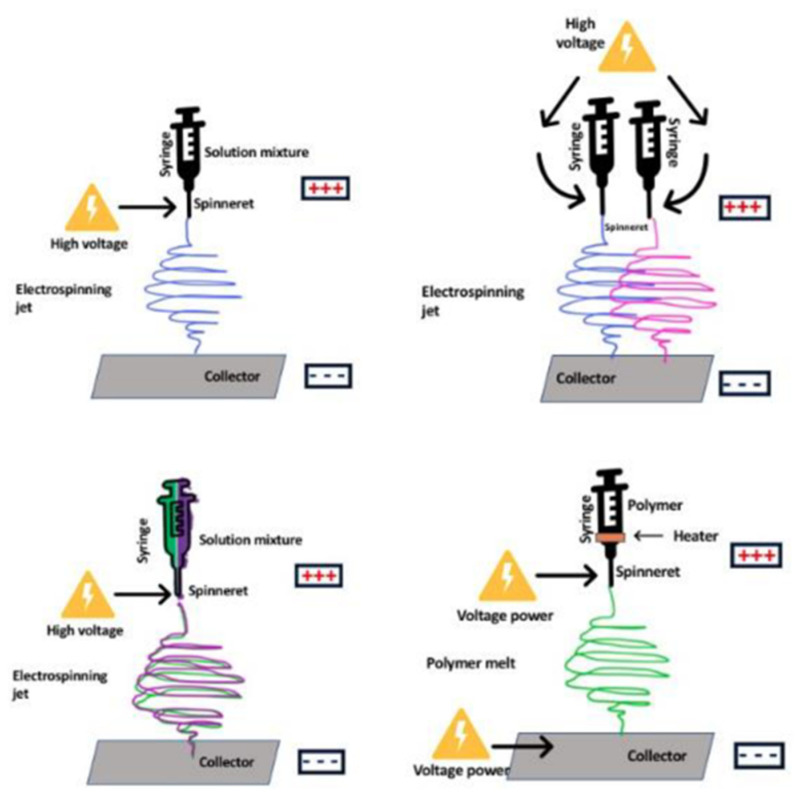
Fabrication processes for electrospun nanofiber composites [201].

Nanoparticles or nanofillers included in the spinning fluid can enhance the properties of electrospun nanocomposite membranes (Figure 5). One study examined the fabrication of composite electrospun fiber membranes with sorptive properties for the removal of heavy metals. The nanosorption capacity of boehmite was investigated as a function of pH and time. Using electrospinning, a composite submicron fiber membrane coated with boehmite nanoparticles was produced. The results indicate that sorption of Cd (II) by a boehmite-impregnated electrospun membrane with a capacity of 0.20 mg/g was feasible [200].

In one study, tiny titanium dioxide (TiO_2_) particles were effectively embedded in nylon-6 spider-net-like nanofiber mats. The antifouling effect, mechanical strength, antibacterial protection, and UV protection were all enhanced by the introduction of a small amount of TiO_2_ NPs to the electrospun mats [202].

Furthermore, MMNMs electrospun in PVDF nanofiber with nanoclay were used in direct contact membrane distillation, and the membrane’s hydrophobicity/hydrophilicity, surface roughness, porosity, and membrane charge were investigated. It appears that clay particles altered the crystallization of the nanocomposite membranes by increasing the melting point of the membranes generated [203].

In another work, electrospinning was used to create nylon-6/silver nanofibers (nylon 6/silver). Using nylon 6/silver as a barrier against *S. aureus* and *K. pneumoniae*, growth was proven to be an effective antibacterial strategy. Antibacterial properties were absent in silver nanoparticle-free nylon 6 fibers. Water filters, wound care, and anti-adhesion membranes might benefit from electrospun nylon 6/silver nanocomposites [204].

Hosseini et al. produced and employed montmorillonite (Mt) chitosan/poly(vinyl alcohol) (PVA) nanocomposite electrospun nanofibrous membranes (ENM) to treat colored wastewater. Mt with varied mass percentages (0, 1.0, 2.0, and 3.0 mass percent) was included in the membrane structure, and its influence on the pore size, morphology, porosity, mechanical strength, and permeation capabilities of ENM was examined. The membranes were employed as affinity screens for dye removal through ultrafast penetrating adsorption. Adding Mt as a strengthening element increased the nanocomposite ENM’s resistance to compaction, as demonstrated by the findings. Young’s modulus rose from 0.89 MPa for chitosan/PVA ENM to 2.09 MPa for nanocomposite membranes containing 1% Mt. It was discovered that the optimal concentration of Mt for increasing both water flow and dye removal was 2.0 mass percent. Effectively resolving the trade-off between permeability and selectivity, the improved membrane repelled 95 percent of Basic Blue 41 (BB41) at a flux of 1765 L/m^2^/h at 0.4 bar working pressure [205].

## 6. Challenges and Perspective

Despite the advances made in the use of nanotechnology-based approaches for water treatment, several obstacles and challenges still need research efforts and considerations to realize the promise of these technologies. A major limitation is the fouling of the nanoadsorptive membrane through agglomeration of extraneous particles in the membrane. This problem can be solved through the introduction of nanoparticles with anti-fouling characteristics, which can be produced by the processing, functionalization, and surface modification of these nanomaterials. Additionally, further approaches can be pursued to avoid the regrowth of microbes on the surface of membranes during water treatment processes. The comparatively poor adsorption capacity of the adsorptive systems is an additional issue requiring more investigation. The membranes’ adsorption capacity is affected by the limited number of adsorbents that can be included into the membrane matrix. To determine the practicality of deploying adsorptive membranes on an industrial level, exhaustive cost–benefit evaluations should be done. Moreover, the technological requirements of using adsorptive membranes on the industrial scale still need further investigation.

In addition, the safety and nontoxicity of the adsorbent materials introduced to the membrane is a critical problem to be solved. Toxic fillers are used in water purification, resulting in contaminated water. There should be no danger to individuals or to the environment associated with any water treatment method used. Multiple tests and experiments can be used to determine the quality of water generated—whether it is acceptable for human consumption or for discharge into the aquatic environment. Moreover, many of the published articles highlighted the effectiveness of the materials and adsorbent systems in removing heavy metals and other pollutants from water and the environment, but they do not sufficiently address the reusability of the contaminants that have been removed from the environment. The idea of reusing the heavy metals and other pollutants removed from industry waste streams should be investigated further.

Furthermore, the development of novel nanomaterials with better functions for adsorptive membranes warrants further research. Even with the evaluation of many materials in the laboratory, there are still drawbacks in finding new materials that could be used in mixed matrix membranes for better adsorption of heavy metals. The high cost of producing these materials is also a significant factor to consider. The possibility of finding and developing novel nanomaterials with affordable cost and superior characteristics that can break into the market for the commercial scale-up of adsorptive membranes should also be researched. Finally, the future outlook in developing nanotechnology-based water treatment solutions is promising, and the advances in the application of these technologies are touted to deliver reliable answers to water treatment and environmental cleanup issues.

## 7. Conclusions

In summary, the use of nanotechnology in many contexts has led to advancements in engineering and environmental research, as well as to the development of new nanotechnology-based technologies. Extremely harmful to individuals and the environment are the high levels of undesired or unrecognized pollutants that make wastewater treatment a global priority. Emerging nanoscale technology has driven the advanced use of new and low-cost techniques, such as nanoadsorptive membrane processes that are suitable for filtration and adsorption procedures for the removal of environmental toxins. In recent years, improvements in membrane qualities such as thermal properties, selectivity, hydrophilicity, and permeability have been made. This can be advanced by the production of more low-cost and efficient polymeric nanocomposite membranes. In addition, it has been established that the incorporation of nanoparticles into the polymer matrix improves the chemical, mechanical, and thermal characteristics. This review focuses on the introduction of several nanomaterials into membranes in diverse water treatment applications.

## Figures and Tables

**Table 1:** Summary of nanoparticles used as adsorbents for water treatment.

Nanomaterial	Example	Particle Size	Absorption Capacity	Pollutants	Ref
(A) Carbon-Based Nanomaterials					
Graphene-based nanomaterials (GNMs):	Graphene oxide (GO) and TiO_2_@rGO nanohybrids	25 nm		Organic solvent	[63]
Carbon nanotubes (CNTs)	Microwave-heated MWCNTs	10–23 nm 18 to 28 nm	104.2 mg/g 99 mg/g	Pb (II) Cu (II)	[64,65]
(B) Metal and Metal Oxide-Based Nanoparticles					
Nanosized iron oxide	Fe_3_O_4_, α-Fe_2_O_3_, μ-Fe_2_O_3_	15.69–85.84 nm	6.33–200 mg/g	Cr(VI) and As(V)	[66]
Nanosized titanium dioxide	TiO_2_	18 nm	333.33 mg/g 250 mg/g	Pb(II) Cd(II)	[67]
Noble metal-based nanoparticles	Au, TiO_2_ NBsa/Au NPs	5–15	-	Tetrabromobisphenol A	[68]
(C) Hydrogels					
Magnetite in polystyrene-co-polymethacrylic acid (PS-co-PMAA)	Fe_3_O_4_, PS-co-PMAA	~100 nm	8.49 to 53.37, 11.17–80.69, and 10.75–65.35 mg/g	Cs^+^, Co^2+^, and Sr^2+^	[69]
(D) Nano-sponges					
Zeolite nanosponges	Ni	3–5 nm	-	Nitrate in contaminated water	[70]
Cyclodextrin-based nanosponges	EDTA-cross-linked β-cyclodextrin	-	1.241 and 1.106 mmol.g^–1^	Cu(II) and Cd(II)	[71]
β-cyclodextrin covalently cross-linked tannic acid	Reduced graphene oxide (RGO), beta-cyclodextrin (βCD), and epichlorohydrin	-	1321.01 mg/g	Cr(VI)	[72]
(E) Nanocomposites					
Magnetic nanocomposites	-	<10 nm	29 to 641 mg/g 333–362 mg/g	Co(II), Ni(II), Cu(II), and Pb(II) Cs(I)	[73]
Mineral-based nanocomposites	Nickel ferrite nanocomposite functionalized with L-cysteine-attached 3-glycidyloxypropyltrimethoxysilane	10–15 nm	122 mg/g, 135 mg/g, and 150 mg/g	Remove the fluoroquinolone class of antibiotics (lomefloxacin, ciprofloxacin, and norfloxacin)	[54]
(F) Layered Double Hydroxide (LDH)-Based Materials					
Mg/Fe-LDO hollow nanospheres	Flower-like Mg/Fe-layered double oxide	17.1 nm	1250 mg/g and 2000 mg/g	Organic dyes: Congo red and methylene blue	[74]
Fe_3_O_4_/graphene oxide/LDH	-	20 nm	-	Pd (II) and 2,4-dichlorophenoxyacetic acid from an aqueous system	[62]

**Table 2:** Nanomaterials for heavy metal removal.

Type of Adsorbent	Shape and Size	Specific Surface Area	Heavy Metals	Removal Rate/Adsorption Capacity	Isotherm Model	pH	Dosage	Reference
AgNP_s_	Spherical, 46.2 nm	-	Pb^2+^, Cr^6+^, Cd^2+^	72.6% 81.3% 88.1%	Freundlich model	8.68	0.75 g	[102]
AgNPs/banana leaf powder composite	Semi-spherical	-	Zn^2+^, Pb^2+^, Fe^3+^	79% 88% 91%	Langmuir model	5 and 6	0.05 to 0.25 g	[134]
AgNP_s_	Spherical	250 m^2^/g	Ni^2+^, Co^2+^	88%	Langmuir model	9 and 7	40 and 50 mg	[135]
AuNPs/ZnO-ZrO_2_ composite	Granular particles	115.03 m^2^/g	As^5+^	88%	Gunary model	10	0.01 g	[136]
CuO nanoparticles	21.6 nm	-	Pb^2+^	84.2%	Freundlich model	2–6	0.1–1.0 g/L	[137]
Polyaniline/itaconic acid/copper oxide nanocomposite	Oval shape, 20 nm	-	Cr^6+^	75–96%	Langmuir and Freundlich models	2–6	0.2–1.0 g	[138]
CuO nanoparticles	Spherical, 150 nm	20 m^2^/g	Pb^2+^, Ni^2+^, Cd^2+^	18% 52% 84%	Freundlich model	6	0.33 g/L	[113]
Diethylene glycol-functionalized Cu_2_O NPs	Quasi-spherical, 57.4 nm	5.35 m^2^/g	Cd^2+^	98%	Langmuir model	6.3	1.0 g/L	[139]
Fe_2_O_3_ nanoparticles	Spherical, 23 nm	-	Cr^6+^, Pb^2+^, Zn^2+^	92.26% 75.57% 89.36%	Thomas, Yoon–Nelson and BDST kinetic models	6	-	[140]
Superparamagnetic Fe_2_O_3_/activate carbon	Spherical, 23–35 nm	-	Cr^6+^	99.7%	Freundlich isotherm model	3	10 g/L	[141]
Chitosan/Fe_2_O_3_/PVDF composite membrane	-	-	Cr^6+^	90.45%	Langmuir model	4	-	[142]
PVDF/PVP/TiO_2_ membrane	-	-	Cu^2+^	96.36%	Freundlich isotherm model	10	1 wt.% TiO_2_	[143]
Acid-activated kaolinite clay/titanium oxide nanocomposite	-	32.98 m^2^/g	Mn^2+^, Fe^3+^, Pb^2+^, Cu^2+^	89.37% 91.99% 81.95% 32.39%	Langmuir model	10	0.5 g	[144]
Cellulose nanocrystals/Ag or ZnO	-	-	Pb^2+^	94%	Langmuir model	2–8	0.05 g	[145]
Al doped ZnO nanoparticles	-	20.76 m^2^/g	F^–^	98%	Temkin isotherm model	7	0.005 g	[146]
ZnO hollow fiber membrane	35–85 nm	-	Cu^2+^	92%	Langmuir model	8	2 wt.%	[147]
MgO/WO_3_ nanocomposites	Spherical	104.2 m^2^/g	Cu^2+^ Fe^3+^ Cr^6+^	98.1% 100% 100%	Langmuir model	-	-	[148]
MgO nanoparticles	Rod shape 30–85 nm (length) and 8.3–16.4 nm (width)	-	Cr^6+^, Co^2+^, Pb^2+^, Cd^2+^, Ni^2+^	94.2%, 63.4%, 72.7%, 74.1%, 70.8%	-	-	100 mg	[149]
MgO nanoparticles	Cubic shape, 25–39 nm	-	PO_4_^3-^	72%	Freundlich isotherm models	12	0.01 g	[148]
Cerium oxide/corncob nanocomposite	-	-	Cd^2+^ Cr^6+^	95% 88%	Intra-particle diffusion model	9	20 mg	[150]
CeO_2_ nanoparticles	Spherical, 10 nm	-	UO_2_^2+^	96%	Langmuir and Freundlich models	4	0.003 mg	[151]
Ce_2_O_3_/SiO_2_/and Ce_2_O_3_/ZnO	Spherical shape	84.62 m^2^/g and 46.12 m^2^/g	Cr^6+^	55% and 50%	Langmuir isotherm model	7	0.02 g	[152]

## Data Availability

Data are contained within the article.
